# 3D-printed porous titanium suture anchor: a rabbit lateral femoral condyle model

**DOI:** 10.1186/s12891-024-07666-w

**Published:** 2024-07-18

**Authors:** Lien-Chen Wu, Yueh-Ying Hsieh, Ting-Shuo Hsu, Po-Yi Liu, Fon-Yih Tsuang, Yi-Jie Kuo, Chia-Hsien Chen, TIN Van Huynh, Chang-Jung Chiang

**Affiliations:** 1 Department of Orthopaedics, Shuang Ho Hospital, Taipei Medical University, New Taipei City, 23561 Taiwan; 2 Department of Orthopaedics, School of Medicine, College of Medicine, Taipei Medical University, Taipei, 110 Taiwan; 3 Graduate Institute of Biomedical Materials and Tissue Engineering, College of Biomedical Engineering, Taipei Medical University, Taipei, 110 Taiwan; 4 Division of Neurosurgery, Department of Surgery, National Taiwan University Hospital, Taipei City, 10022 Taiwan; 5 Spine Tumor Center, National Taiwan University Hospital, Taipei City, 10022 Taiwan; 6 Department of Orthopedic Surgery, Wan Fang Hospital, Taipei Medical University, Taipei City, 116 Taiwan; 7 School of Biomedical Engineering, College of Biomedical Engineering, Taipei Medical University, Taipei, 110 Taiwan; 8 Department of Interventional Cardiology, Thong Nhat Hospital, Ho Chi Minh City, Vietnam; 9 International Ph.D program in medicine, college of medicine, Taipei medical university, Taipei, Taiwan

**Keywords:** Suture anchor, 3D-printed porous anchor, Bone ingrowth, Bone formation

## Abstract

**Background:**

The inclusion of a connecting path in a porous implant can promote nutrient diffusion to cells and enhance bone ingrowth. Consequently, this study aimed to evaluate the biomechanical, radiographic, and histopathological performance of a novel 3D-printed porous suture anchor in a rabbit femur model.

**Methods:**

Three test groups were formed based on the type of suture anchor (SA): Commercial SA (CSA, Group A, *n* = 20), custom solid SA (CSSA, Group B, *n* = 20), and custom porous SA (CPSA, Group C, *n* = 20). The SAs were implanted in the lateral femoral condyle of the right leg in each rabbit. The rabbits (New Zealand white rabbits, male, mean body weight of 2.8 ± 0.5 kg, age 8 months) underwent identical treatment and were randomized into experimental and control groups via computer-generated randomization. Five rabbits (10 femoral condyles) were euthanized at 0, 4, 8, and 12 weeks post-implantation for micro-CT, histological analysis, and biomechanical testing.

**Results:**

At 12 weeks, the CPSA showed a higher BV/TV (median 0.7301, IQR 0.7276–0.7315) than the CSSA and CSA. The histological analysis showed mineralized osteocytes near the SA. At 4 weeks, new bone was observed around the CPSA and had penetrated its porous structure. By 12 weeks, there was no significant difference in ultimate failure load between the CSA and CPSA.

**Conclusions:**

We demonstrated that the innovative 3D-printed porous suture anchor exhibited comparable pullout strength to conventional threaded suture anchors at the 12-week postoperative time-point period. Furthermore, our porous anchor design enhanced new bone formation and facilitated bone growth into the implant structure, resulting in improved biomechanical stability.

## Introduction

3D-printed metallic implants have been proposed as alternatives to traditional solid structures because of the lower stress shielding and better bone ingrowth [1–3]. The ability to print detailed complex structures that can withstand physiological loading has allowed 3D printing to be adopted into orthopedics, such as for spinal intervertebral implants, surgical guides, and other bone implants [1–4]. In additional, incorporating a porous structure in the implant allows for bone tissue adherence and ingrowth. Previous studies have shown that a pore size of 200–600 μm is suitable for osteoid formation and mineralization, while 50–100 μm pores can improve vascularization and nutrient diffusion during bone reconstruction [5–8]. To ensure long-term survival, the design of bone implants with porous structures must also consider the elastic modulus, static and dynamic strength, and wear and corrosion resistance [9].

Clinically, implant loosening and migration are the most common complications with metallic suture anchors [10]. Our previous study developed a 3D-printed rectangular titanium anchor with porosity of 70% and pore sizes of 600 μm, and the initial fixation of the implant in bone is created by friction [11]. Our implant is a hybrid device which acts as both a fixation device and scaffold and has an initial fixation strength comparable to a metallic threaded anchor.

While the porous titanium anchor developed in our previous research had a sufficient initial fixation strength, the bone regeneration efficiency and long-term fixation strength are still unknown. Studies have shown that an inadequate pore size or structures with insufficient connection paths cannot achieve effective bone ingrowth [12, 13]. Animal models are considered as an invaluable tool in evaluating the functionality of implants during the bone growth stage [14, 15]. Therefore, we created a rabbit model to verify the impact of bone growth on our novel implant. We considered that porous implants with connecting paths could allow adequate diffusion of nutrients to cells and improve bone ingrowth to the implant. Based on this concept, using a porous implant combined with the bone tissue postoperatively may improve the fixation strength after long-term implantation. The purpose of this study was to evaluate the anchor fixation effectiveness and feasibility of a press-fit connected pore structure scaffold by assessing its biomechanical, radiographic, and histopathological performance in a rabbit femur model.

## Materials and methods

### Production of anchors

**Fig. 1:**
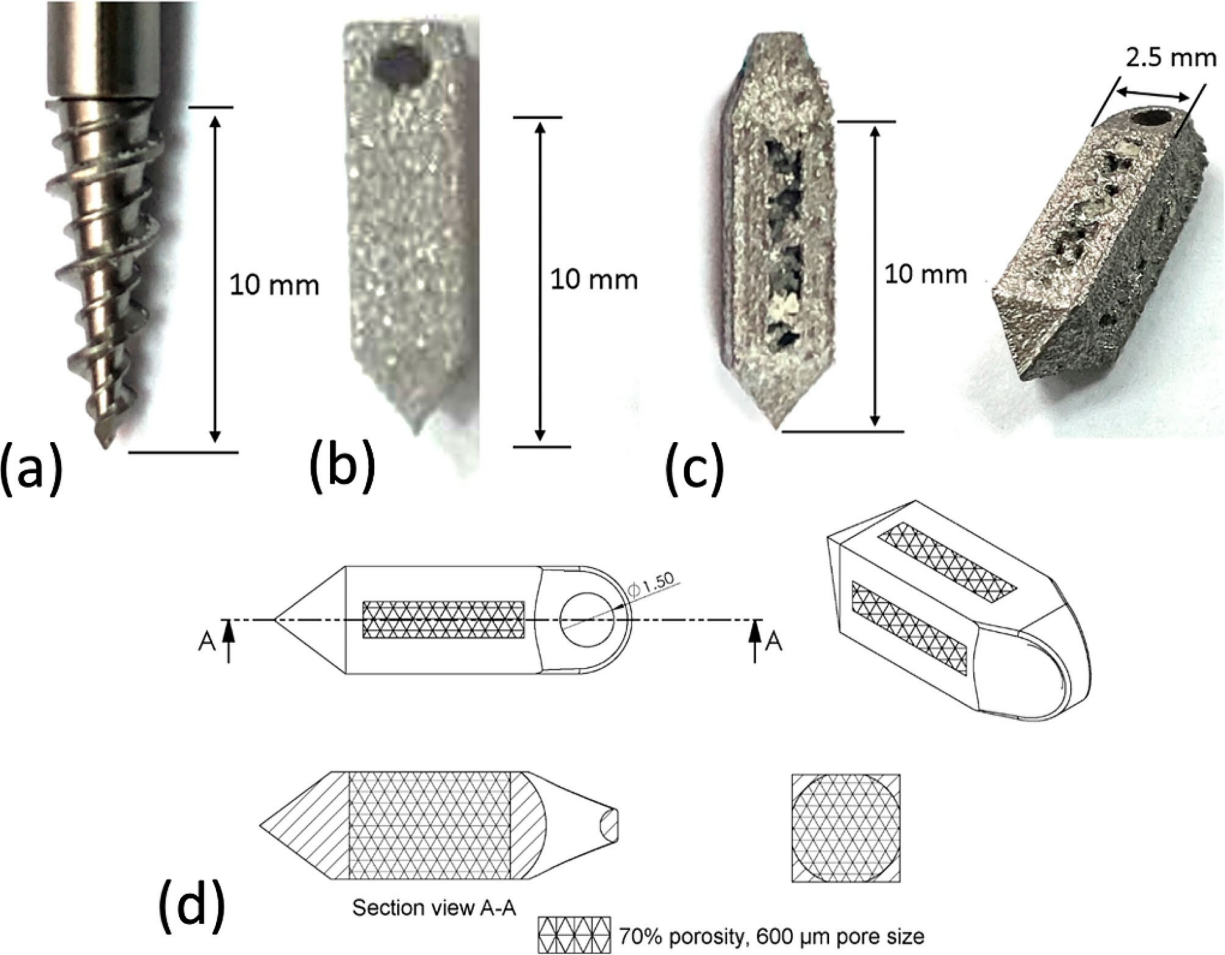
(**a**) Commercially-available threaded suture anchor (TwinFix™ Ti 3.5, Smith & Nephew, Andover, MA, USA); (**b**) custom-made threadless suture anchor with rectangular cross-section solid construction; (**c**) custom-made threadless suture anchor with rectangular cross-section porous construction and (**d**) design drawing

Three types of suture anchor (SA) were assessed in this study: (type i, CSA) a commercial threaded titanium anchor with an outer diameter of 3.5 mm and a length of 10 mm (TwinFix™ Ti 3.5, Smith & Nephew, Andover, MA, USA, Fig. 1a); (type ii, CSSA) a custom-made, 3D-printed titanium alloy suture anchor with a rectangular cross-section and solid construction (Fig. 1b), measuring 2.5 mm × 2.5 mm × 10 mm (W × D × H); and (type iii, CPSA) a custom-made, 3D-printed titanium alloy suture anchor with a rectangular cross-section, surface fenestrations and a porous body (Fig. 1c and d). The custom SAs were designed using Creo 2.0 (PTC, Boston, MA, USA) and the CAD models were used to 3D print the anchors using EOS Titanium Ti64ELI powder with an EOS M290 Selective Laser Melting Metal Additive Manufacturing Machine (EOS GMbH, Krailling, Germany). We utilized the recommended machine parameters provided by the equipment manufacturer, including a laser power of 280 W, scan speed of 1200 mm/s, resolution of 0.02 mm, and layer thickness of 30 μm.

### In vivo animal study design

All animal experiments were approved by the Ethics Committee of the Master Laboratory Co., Ltd in accordance with national animal welfare legislation (approval no.: IACUC-22T10-33) and adhered to the Animal Research: Reporting In Vivo Experiments (ARRIVE) criteria. A total of 60 male New Zealand white rabbits (provided by the local Animal Center) with a mean body weight of 2.8 ± 0.5 kg and age 8 months were used for this study. New Zealand white rabbits achieve growth plate closure of the femur and tibia after 4.8–5.8 months and 5.5-8 months, respectively [16]. All animal handling procedures, including housing and husbandry conditions, animal care, and monitoring, were approved by the Taiwan Centers for Disease Control and adhered to the Guidelines for the Care and Use of Laboratory Animals established by the Taiwan Council of Agriculture. The rabbits were divided into three groups for the three SA designs under test: TwinFix Ti 3.5 anchor (CSA) (Group A, *n* = 20); custom-made threadless suture anchor with rectangular cross-section solid construction (CSSA) (Group B, *n* = 20); and custom-made threadless suture anchor with rectangular cross-section and porous construction (CPSA) (Group C, *n* = 20). Each rabbit was randomized into an experimental and control group using a computer-generated randomization method. All rabbits underwent the same treatment procedure, and 5 rabbits (10 femoral condyles) in each group were euthanized immediately after implantation for micro-CT/histological analysis and biomechanical testing. The other rabbits were euthanized after implantation periods of 4 weeks, 8 weeks, and 12 weeks after surgery (5 femoral condyles in each group) for micro-CT/histological analysis and biomechanical testing. All animals were euthanized with an intravenous injection of pentobarbital sodium (200 mg/kg) through the ear vein to ensure painless euthanasia.

### Surgical methods

All surgical procedures were conducted under general anesthesia administered by intramuscular injection of a mixture of Zoletil and Rompun (Zoletil 15 mg/kg; Rompun 0.05 mL/kg; Zoletil, Virbac Taiwan, Taipei, Taiwan; Rompun, Bayer Taiwan, Taipei, Taiwan). Analgesia was administered to all rabbits by intramuscular injection of ketoprofen (2 mg/kg, ASTAR, Hsinchu, Taiwan) 24 h prior to the operation, and this continued for 7 consecutive days post-surgery. As a prophylactic measure against infection, the rabbits received intramuscular gentamycin (5 mg/kg, Standard Chem. & Pharm, Tainan, Taiwan) 24 h before the operation and continued this treatment for 7 consecutive days after the surgery.

**Fig. 2:**
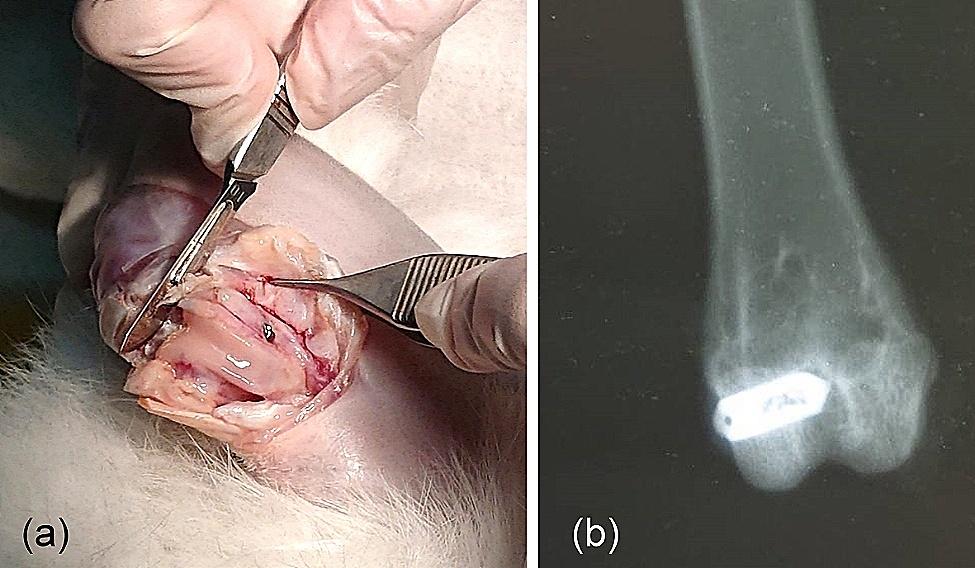
(**a**) Image to demonstrate the exposure of the lateral femoral condyle, location of the drilled hole and SA inserted along the axis of the hole. (**b**) The lateral femoral condyle was the site where the SA was inserted, as indicated by the radiograph

A lateral excision was created to expose the lateral femoral condyle of the right leg. A transverse hole in the bone (10 mm in depth) was drilled and then dilated with sharp and blunt broaching bits (2.5 mm in diameter; Fig. 2). The implant was then placed into the hole. The incision site was closed with sutures. X-ray examinations were performed immediately after the operation, and 2 × 10^5^ IU of penicillin was intramuscularly injected once per day until the third postoperative day [17].

### Micro-CT analysis

After euthanizing the rabbits, five specimens were extracted from each group and multi-scale nano-CT (Skyscan 2211, Bruker Micro-CT, Kontich, Belgium) was utilized with a voxel resolution of 30 μm, a voltage of 155 kVp, a current of 80 µA, and a 6 W output in micro-focus mode with a 360° scan. Image reconstruction was performed using InstaRecon xCBR (version 2.0.4.6, InstaRecon, Champaign, IL, USA) and NRecon (Bruker Micro-CT, Kontich, Belgium). NRecon was also used for correcting ring artifacts and beam hardening.

**Fig. 3:**
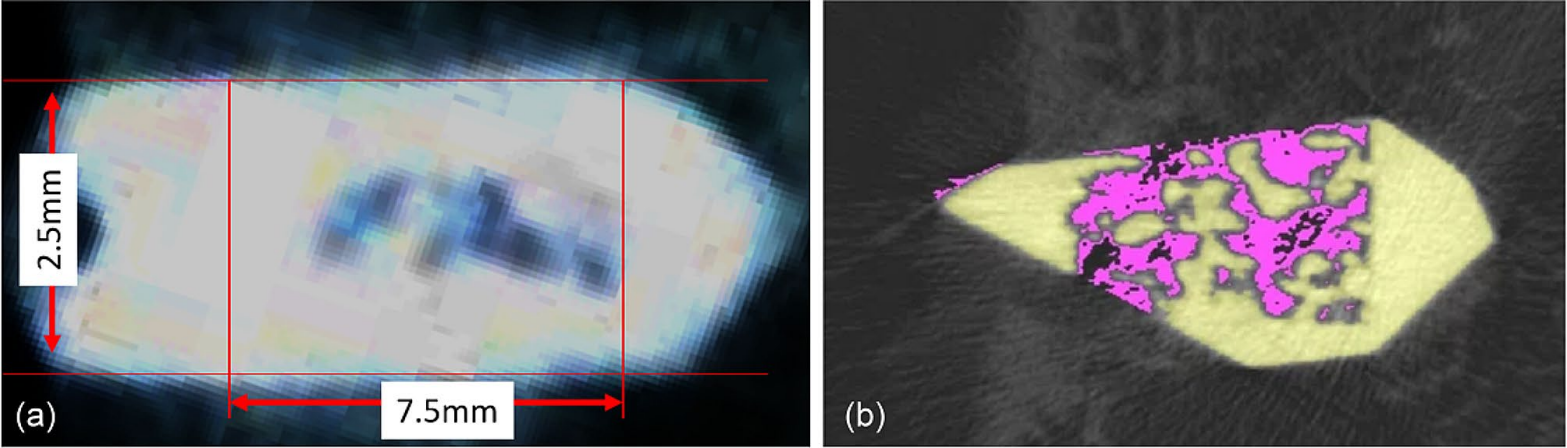
(**a**) The reconstructed cross-sections were re-orientated, and the region of interest (ROI) was further selected. (**b**) Automatic Ostu thresholding and bone ingrowth analysis were performed using CTAn software

To reposition the reconstructed cross-section and define the region of interest (ROI), we isolated a region from the proximal region of the suture hole to the distal region of the anchor tip column. We conducted the analysis using 2.5 mm (100 slices) images. The CTan (Bruker, Belgium) was utilized for automated Otsu thresholding and bone growth analysis. We designated a region around the implant spanning 200–1000 μm as the ROI for bone ingrowth analysis (Fig. 3). Bone and metal anchors were differentiated by disparities in X-ray absorption. We utilized the CTAn software, incorporating a shrink-wrap algorithm, to delineate the boundary of the metallic anchors. The bone volume (BV, mm3) within the 200–1000 μm ROI of the metallic implant was calculated. For 3D visualization, we employed Avizo software (Thermo Fisher Scientific, MA, USA) and CTVox (Bruker Micro-CT, Kontich, Belgium).

### Histological analysis

Five specimens were used from each group for histological analysis. The samples were initially fixed in 10% formalin for 14 days, followed by sequential dehydration using increasing concentrations of ethanol (70%, 95%, and 100%) for a minimum of 1 day. The samples were then infiltrated with polymethylmethacrylate for 5 days [18, 19]. After embedding, the samples were cut vertically, perpendicular to the long axis of the CPSA, at the level of the bone-implant interface. Upon polymerization, non-decalcified Sect. (500 μm thick) were created by transversely sectioning the specimens using a microtome equipped with a diamond blade. These sections were subsequently further thinned for histological staining. Masson’s trichrome stain was applied to the sections and the bone-implant interfaces were examined with a light microscope (Nikon Eclipse Ti-series, Melville, NY, USA).

### In-vitro biomechanical analysis

**Fig. 4:**
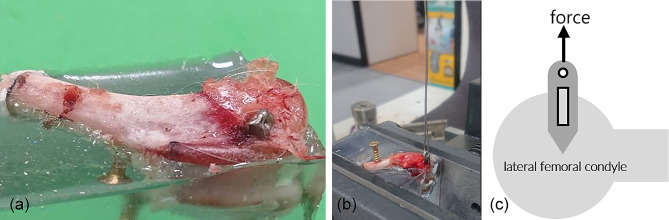
(**a**) The specimens were uniformly embedded into the agglomerating mixture. (**b**) The suture was passed through the eyelet at the top of the SA. The SA will be pulled out of the lateral femoral condyle during the mechanical test. (**c**) The direction of force application is along the long axis of SA.

The femoral condyles were truncated below the level of the greater trochanter. Aluminum boxes measuring 2 cm × 2 cm × 5 cm were fabricated. Self-curing denture acrylic and liquid denture acrylic were poured into the boxes and mixed together. The specimens were uniformly embedded within the mixture. Special care was taken to shield the head parts of the prostheses by using plasticine to prevent them from adhering to the denture acrylic mixtures and the prostheses were maintained in a vertical alignment. Once the mixtures had solidified, the aluminum boxes were removed. Each specimen was secured in an MTS 858 mini Bionix II instrument and subjected to pull-out testing. An initial preload of 1 N was applied to each specimen, followed by a test load at a rate of 5 mm/min acting parallel to the axis of the anchor insertion into the femoral condyle (Fig. 4). The maximum forces needed to extract the prostheses from the bone were recorded.

### Statistical analysis

All experimental data are presented as the median and interquartile range (IQR). Nonparametric analysis was conducted using the Wilcoxon rank-sum test. A p-value of < 0.05 was considered statistically significant. Statistical analysis was performed using SPSS Statistics (version 26, Chicago, IL, USA).

## Results

### Micro-CT analysis

**Fig. 5:**
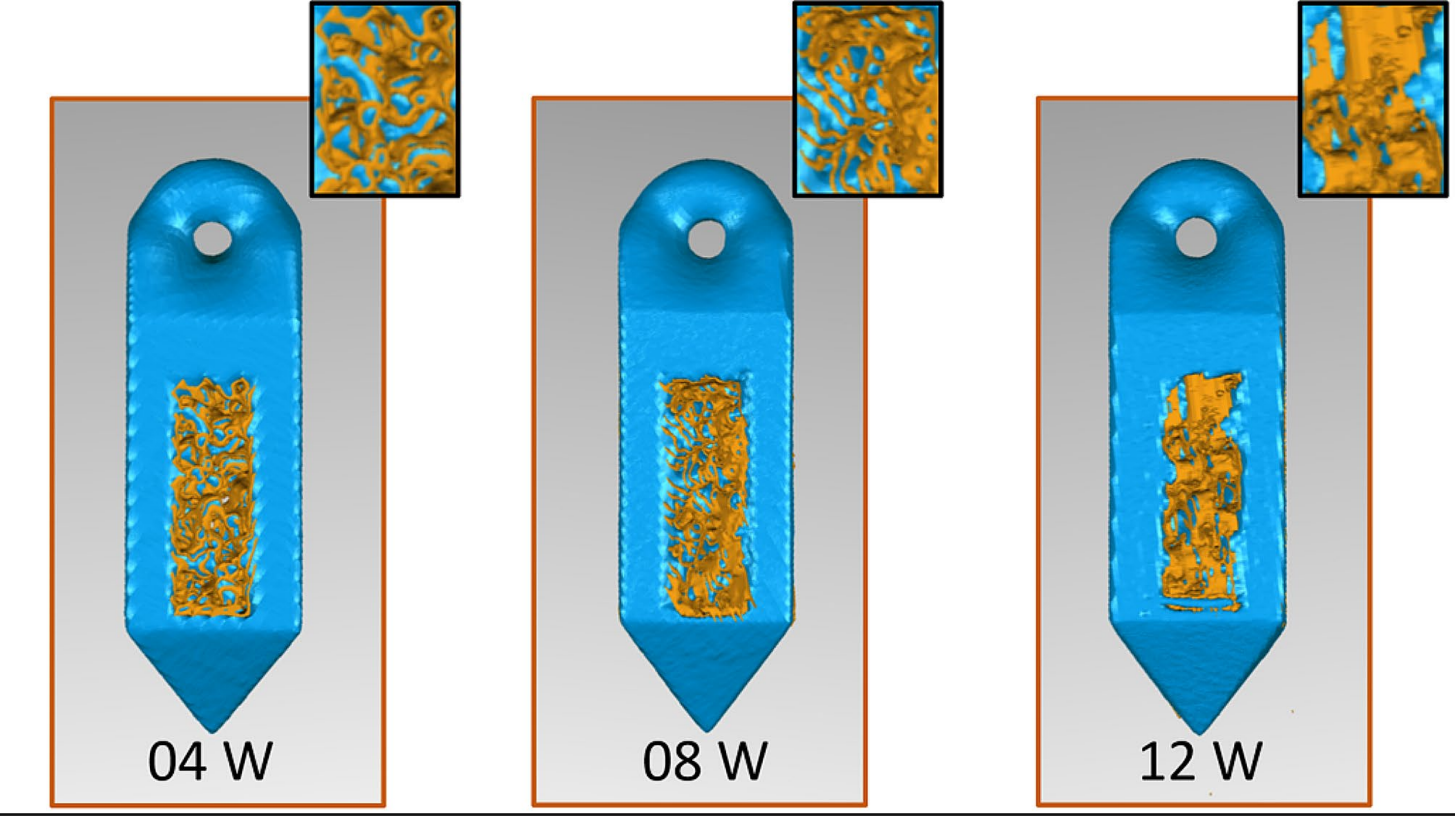
Micro-CT 3D reconstruction of the bone-implant interface was performed. After 4, 8, and 12 weeks of bone healing, images from the micro-CT reconstruction indicated bone formation within the CPSA implants

Micro-CT was used to evaluate bone ingrowth into the porous SA. The CPSA exhibited a higher postoperative BV/TV at 12 weeks (0.7301, 0.7276–0.7315) than CSA and CSSA (Table 1). The CPSA exhibited a lower postoperative BV/TV at 4 and 8 weeks (0.3742, 0.3701–0.3762 vs. 0.5100, 0.5077–0.5114) than CSA and CSSA. There was no difference in the BV/TV intra-group analysis between the 4- and 8-week samples in the CSSA and CPSA groups. Figure 5 shows the Micro-CT 3D reconstruction of the CPSA and bone interface after 4, 8 and 12 weeks implantation, indicating bone formation within the CPSA implants.

**Table 1:** BV/TV ratio among the three types of suture anchor (SA) at 4, 8 and 12 weeks

	Median [IQR]		
	BV/TV of CSA	BV/TV of CSSA	BV/TV of CPSA
4 weeks	0.3807 [0.3781–0.3814]	0.3790 [0.3755–0.3799]	0.3742 [0.3701–0.3762]
8 weeks	0.5204 [0.5180–0.5215]	0.5500 [0.5484–0.5510]	0.5100 [0.5077–0.5114]
12 weeks	0.5901 [0.5878–0.5913]	0.6101 [0.6079–0.6113]	0.7301 [0.7276–0.7315]

### Histological analysis

**Fig. 6:**
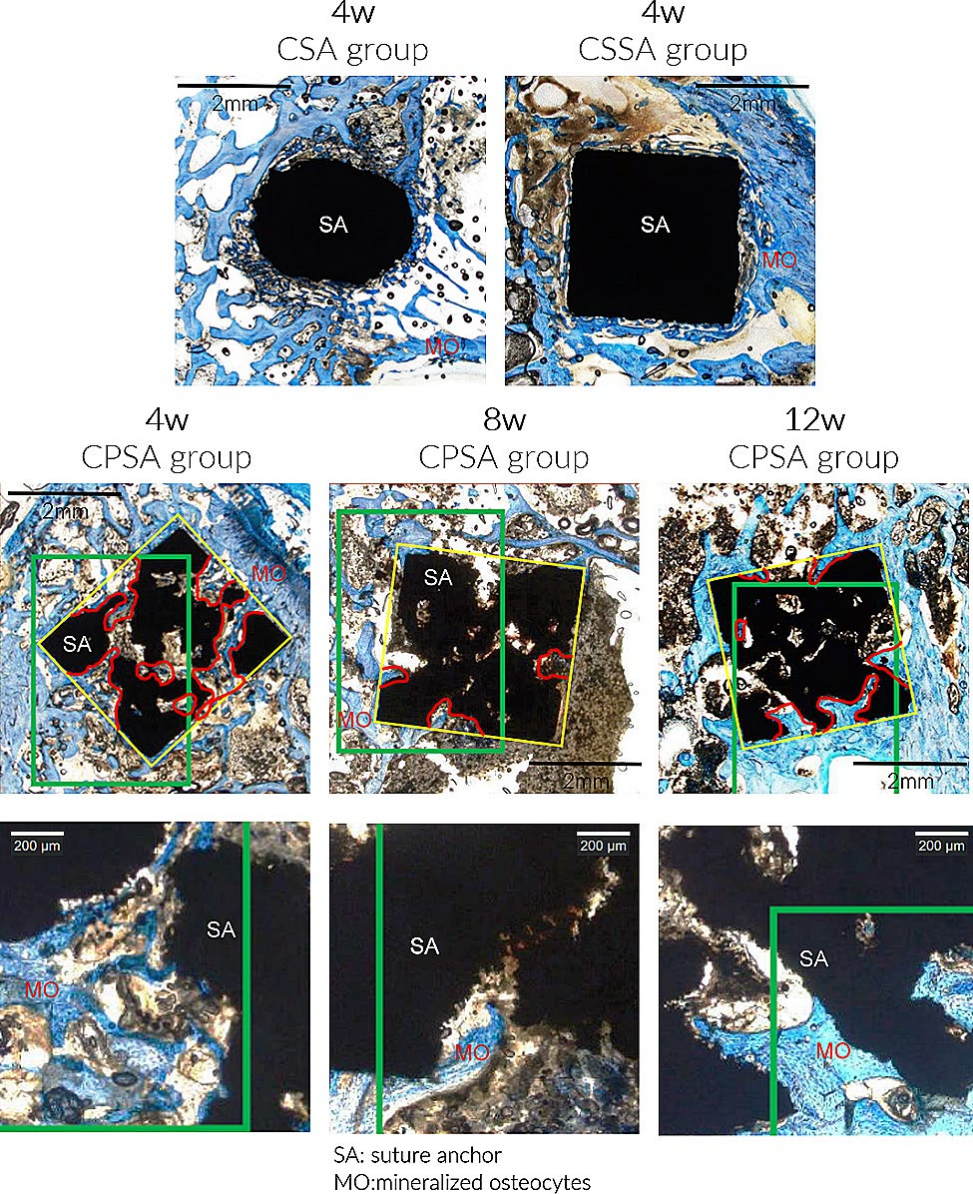
Micro-CT and histological examination of the bone–SA interface 4, 8 and 12 weeks postoperation

Mineralized bone formation was observed in all groups after 4, 8 and 12 weeks (Fig. 6). Mineralized osteocytes were also observed in all groups in bone regions that closely contacted the SA. At 4 weeks, new bone formation was observed around the CPSA and the bone penetrated the porous structure.

### In vivo biomechanical analysis

**Fig. 7:**
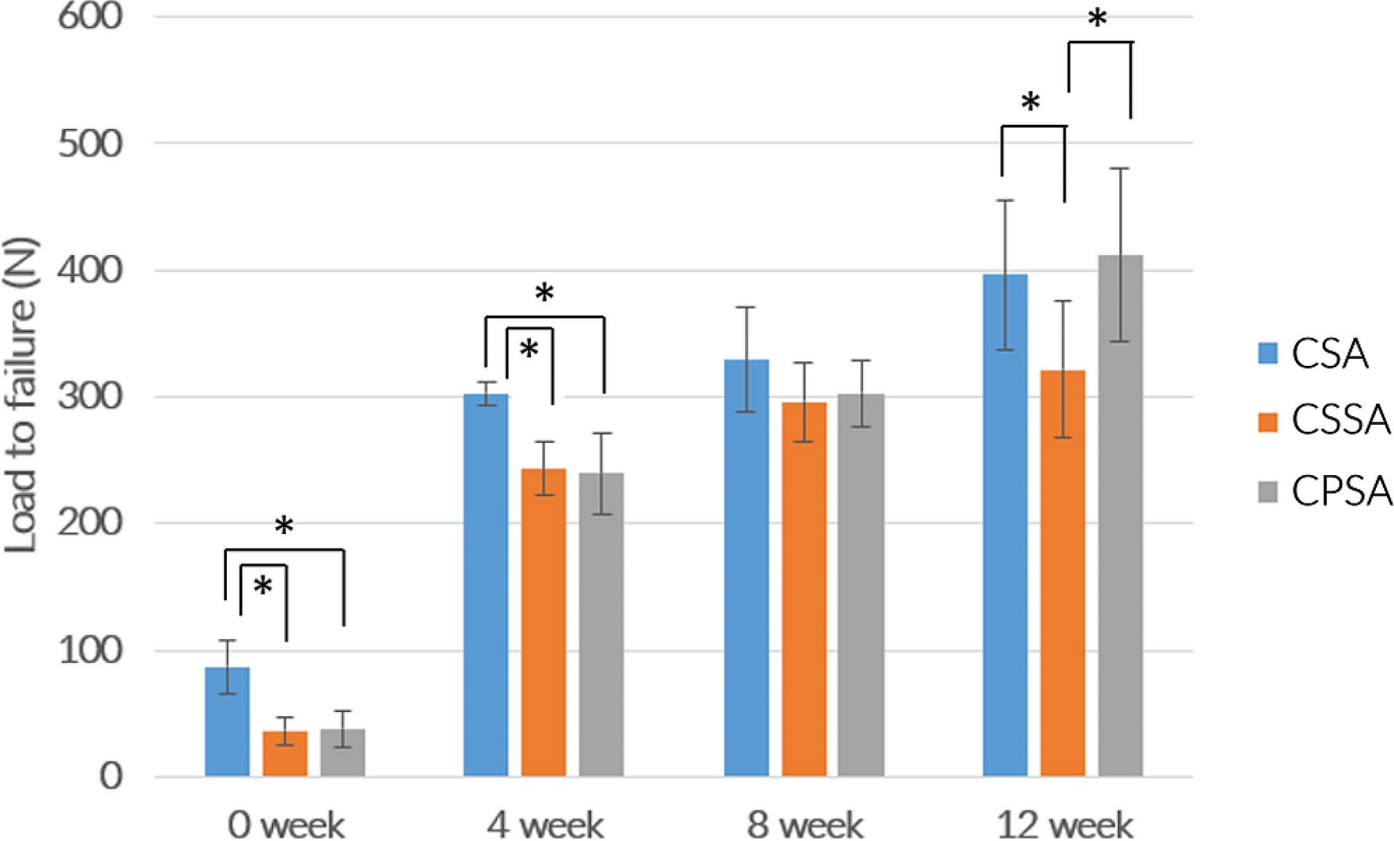
In-vitro biomechanical ultimate pullout strength assessment for different SAs at 0, 4, 8, and 12 weeks after surgery. Median [IQR]. * *p* < 0.05

The difference in ultimate failure load between the CSSA and CPSA at 0 weeks (38, 31–41 N and 39, 31–44 N, *p* = 0.42, respectively) and 4 weeks (235, 228–251 N and 236, 210–262 N, *p* = 0.41, respectively) was not significant. At 8 weeks, there was no significant difference in the ultimate failure load between all groups (Table 2; Fig. 7). At 12 weeks, there was no significant difference in the ultimate failure load between CSA and CPSA (361, 355–442 N and 374, 369–439 N, *p* = 0.35, respectively).

**Table 2:** The ultimate failure load of each SA group at different time points

SA group	Load to failure (Median [IQR])			
	0 week	4 week	8 week	12 week
CSA	88 [75–95]	305 [301–309]	319 [298–351]	361 [355–442]
CSSA	38 [31–41]	235 [228–251]	299 [287–313]	321 [286–342]
CPSA	39 [31–44]	236 [210–262]	302 [288–312]	374 [369–439]

## Discussion

Suture anchors have become a routine tool for securing soft tissue to bone in orthopedic operations. The stability of the anchors depends on the holding capacity of the anchor in the bone. Despite reported successes with suture anchor fixation, loosening of the screw-shaped anchors is still a common complication that can lead to inadequate healing of soft tissue and may require revision surgery [20, 21]. Poor bone–screw integration at the interface on the screw surface may be a significant factor in suture anchor loosening. Bone ingrowth into the implant could potentially enhance the long-term stability of suture anchors, as fixation strength is expected to improve during the bone healing period. Therefore, we developed a 3D-printed scaffold for bone-tissue regeneration in suture anchor applications [11]. In this study, we observed new bone formation and bone ingrowth into the 3D-printed scaffold, resulting in increased mechanical fixation stability [11]. 

The radiographs showed gradual new bone formation over time around the CSSA and CPSA. The rough surface of the anchor may be a major factor facilitating fibroblast and osteoblast adhesion [22–24]. Tsai et al. [23] reported a porous titanium alloy screw could provide better bone-tendon-implant integration than traditional screws. With the CPSA, after 12 weeks the total bone volume fraction was higher than the volume at 8 weeks. Bones typically take 6–12 weeks to undergo substantial growth. Hong et al. [25] reported that a titanium alloy porous implant formed a good integration with bone after 12 weeks, which was comparable to our findings.

Previous studies have shown that Ti6Al4V has excellent biocompatibility when used for orthopedic implants, but its high stiffness and rigidity limits the clinical applications. This current study used 3D printing technology to construct a suture anchor with a press-fit fixation to secure the implant in the bone to reduce relative motion between the bone and implant. From the micro-CT reconstruction, the CPSA had bone formation widely distributed around the surface of implant at week 4, 8, and 12. There was substantial growth of new bone within the macropores and interconnected pores of the implant at week 12. Using a similar design concept, Tsai et al. [23] found more bone growth into an interconnected porous screw than into a traditional solid metal screw within 12 weeks after implantation. Our findings were also compatible to previous reports showing that interconnected porous paths increase implant-bone integration [22, 26].

Pullout of loose implants is one of the main causes of failure for suture anchors. This failure mode is often observed at the anchor-bone interface during rotator cuff repair [27], resulting in pullout of the anchor from the bone. In our study, the pull-out strength in the control group (CSA) was stronger than other groups immediately after surgery and at 4 weeks postoperative. At 8 weeks postoperative, the three groups had similar pullout strength. But after 12 weeks, the pullout strength of the CSA and CPSA were significantly higher than CSSA. There was a large increase in the pullout strength of the CPSA between weeks 8 and 12, which is likely because of bone growth into the implant structure. As expected, the CPSA exhibited new bone formation into the implant structure, and the new bone filled the connecting path within the implant after 12 weeks. The pullout strength of the CSSA and CPSA was significantly lower than CSA after 4 weeks because of the lower bone ingrowth. We considered that the press-fit technique and connected pore structure of our design could offer higher permeability for bone tissue, thereby improving the amount of bone ingrowth and enhancing the interface anchorage between bone and implant.

This study has some limitations. The rabbit model may not be truly representative of human physiological conditions. Although no anchor cracking was observed on the radiographic images, this study did not test the fatigue strength of the implants. The fatigue strength is an important factor for implants that undergo cyclic loading during patients’ daily activities. Additionally, longitudinal sections were not included in the histopathological investigation; we only performed cross-sections to evaluate the bone mass around the implant. Also, the implants in this study were recovered after 12 weeks, which may not indicate the long-term mechanical performance. However, in addition to a biomechanical evaluation, the purpose of this study was to assess the radiographic and histopathological performance of a novel 3D-printed porous suture anchor. This objective was achieved with the tests performed. Because the tested groups and analyzed parameters were limited in our study, we have only observed the effects of the press-fit technique and the existing porous structure on bone ingrowth. In future studies, we will analyze parameters such as different porosity characteristics, different bone densities, and the press-fit technique to improve this design.

## Conclusion

Using an animal model, our study showed that the novel 3D-printed porous suture anchor had a comparable pullout strength to conventional threaded suture anchors at 12 weeks postoperative time-point period. In addition, our porous anchor design improved new bone formation and bone growth into the implant structure to achieve biomechanical stability.

## Data Availability

All data generated or analyzed during this study are included in this published article or can be made available from the corresponding author on reasonable request.

## Abbreviations

SA: suture anchor
CSA: Commercial SA
CSSA: custom solid SA
CPSA: custom porous SA
IQR: interquartile range
